# Supplementary material for: Bluetongue Virus Infection of Goats: Re-Emerged European Serotype 8 vs. Two Atypical Serotypes
Source: Viruses. 2022 May 13;14(5):1034. doi: 10.3390/v14051034 (PMC9144285; doi:10.3390/v14051034)
Supplement: Supplementary file 1 [file viruses-14-01034-s001.zip › viruses-1672937-supplementary.pdf]

Table S1: Comparative evaluation of pan-BTV-S10-OIE assay and the modified BTV-S10-primer-probe-mix-v2

**pan-BTV-S10-OIE assay**	
https://www.oie.int/fileadmin/Home/eng/Health_standards/tahm/3.01.03_BLUETONGUE.pdf	
**Oligo ID**	**Sequence (5`- 3`)**
**Orru_BTV_IVI_F2**	tgg aya aag cra tgt caa a
**Orru_BTV_IVI_R2**	acr tca tca cga aac gct tc
**Orru_BTV_IVI_FAM**	FAM-ARG CTG CAT TCG CAT CGT ACG C-BHQ1

**BTV-S10-primer-probe-mix-v2**	
Oligo ID	Sequence (5`- 3`)
**Orru_BTV_IVI_F2**	tgg aya aag cra tgt caa a
**Orru_BTV_IVI_R2**	acr tca tca cga aac gct tc
**Orru_BTV_IVI_FAM**	FAM-ARG CTG CAT TCG CAT CGT ACG C-BHQ1
**Orru_BTV_IVI_FAM_v2**	FAM-AGG CTG CAT ACG CAT CRT ACG C-BHQ1

			BTV-S10-primer-probe-mix-OIE	BTV-S10-primer-probe-mix-v2	ΔCq OIE and V2	BTV-S10-primer-probe-mix-OIE	BTV-S10-primer-probe-mix-v2
**Virus/sample ID**	**matrix**	**dilution**	Ct-value			End RFU	
BTV-30/MNG2-2016_V241.2	cell culture propagated virus diluted in EDTA-blood from cattle	10^{-0}	17,21	15,31	1,9	1251	3264
		10^{-1}	20,59	18,88	1,71	1208	3438
		10^{-2}	23,96	22,33	1,63	1185	3208
		10^{-3}	27,94	25,96	1,98	1044	3155
		10^{-4}	31,74	29,06	2,68	963	2976
		10^{-5}	35,56	31,79	3,77	754	2880
		10^{-6}	37,18	35,55	1,63	729	2225
		10^{-7}	no Cq	no Cq	no Cq	1,67	4,99
BTV-25/GER2018_V247.3	cell culture propagated virus diluted in EDTA-blood from cattle	10^{-0}	14,47	14,31	0,16	3857	4854
		10^{-1}	17,57	17,46	0,11	3989	4803
		10^{-2}	21,01	20,57	0,44	3831	4827
		10^{-3}	24,28	23,84	0,44	4051	4732
		10^{-4}	27,77	28,06	-0,29	3853	4306
		10^{-5}	30,98	30,47	0,51	3462	4171
		10^{-6}	34,31	34,3	0,01	2864	3325
		10^{-7}	37,05	no Cq	no Cq**	2016	4,35
BTV-25/GER2018_V239.2	cell culture propagated virus diluted in EDTA-blood from cattle	10^{-0}	14,08	13,48	0,6	2899	4703
		10^{-1}	17,36	16,97	0,39	2950	4701
		10^{-2}	21,05	20,24	0,81	3065	4705
		10^{-3}	24,35	25,17	-0,82	2905	4524
		10^{-4}	28,72	27,94	0,78	2631	4259
		10^{-5}	31,8	30,45	1,35	2367	3902
		10^{-6}	34,08	33,92	0,16	2241	3061
		10^{-7}	no Cq	no Cq	no Cq	2	2,44

			BTV-S10-primer-probe-mix-OIE	BTV-S10-primer-probe-mix-v2	ΔCq OIE and V2	BTV-S10-primer-probe-mix-OIE	BTV-S10-primer-probe-mix-v2
**Virus/sample ID**	**matrix**	**dilution**	Ct-value			End RFU	
BTV-27v02/FRA2014	cell culture propagated virus diluted in EDTA-blood from cattle	10^{-0}	20,44	20,07	0,37	3010	4323
		10^{-1}	23,89	23,37	0,52	2819	4652
		10^{-2}	27,16	26,59	0,57	2917	4549
		10^{-3}	30,5	30,04	0,46	2708	4276
		10^{-4}	34,89	33,57	1,32	2253	3668
		10^{-5}	37,55	35,88	1,67	1622	2830
		10^{-6}	no Cq	no Cq	no Cq	-0,856	1,42
		10^{-7}	no Cq	no Cq	no Cq	3,57	5,46
BTV-8/GER2007_BH 01/07	cell culture material	10^{-0}	17,21	17,16	0,05	4076	5072
BTV-25_Toggenburg BH 1/13_4	EDTA-blood	10^{-0}	29,36	27,82	1,54	2314	3881
BTV-25_Toggenburg like BH66/18-1	EDTA-blood	10^{-0}	26,53	26,05	0,48	3013	4409
BTV-25_Toggenburg like BH66/18-2	EDTA-blood	10^{-0}	34,59	33,82	0,77	1692	2655
BTV-26/KUW2010/02	cell culture material	10^{-0}	17,98	17,87	0,11	4849	5595
BTV-27v01/FRA2014	cell culture material	10^{-0}	14,81	14,87	-0,06	3970	4401
BTV-30/MNG2-2016_BH38/18-87	EDTA-blood	10^{-0}	29,71	29,5	0,21	3924	4674
BTV-27v03/FRA2014	cell culture material	10^{-0}	15,9	15,91	-0,01	3690	4170
BTV-28/ISR2014/1537/14	cell culture material	10^{-0}	16,95	17,07	-0,12	3324	3515
BTV-35/MNG1-2018_V134	cell culture material	10^{-0}	17,65	17,6	0,05	3973	4729
BTV-35/MNG1-2018_V133	cell culture material	10^{-0}	25,72	25,86	-0,14	3905	4595
BTV-30/MNG2-2016_BH38/18-103	EDTA-blood	10^{-0}	28,61	28,54	0,07	3882	4702
BTV-33-MNG3/2016_V82	cell culture material	10^{-0}	20,6	20,42	0,18	4425	5572
BTV-30/MNG2-2016_BH 83/16_30	cell culture material	10^{-0}	26,61	25,83	0,78	1706	3562
BTV-8/CHE2018_V250.1	cell culture material	10^{-0}	25,61	25,4	0,21	4167	4694
